# P-1996. Performance Evaluation of an Artificial-Intelligence-Driven Single-Cell Imaging Platform for Rapid Phenotypic Antimicrobial Susceptibility Testing

**DOI:** 10.1093/ofid/ofaf695.2161

**Published:** 2026-01-11

**Authors:** Dong Woo Shin, Yunsang Choi, Kyunghwa Lee, Seong Jin Choi, Song Mi Moon, Eu Suk Kim, Hong-Bin Kim, Jeong Su Park, Kyoung-Ho Song

**Affiliations:** Seoul National University Bundang Hospital, Seoul National University College of Medicine, Seongnam-si, Kyonggi-do, Republic of Korea; Seoul National University Bundang Hospital, Seoungnam-si, Kyonggi-do, Republic of Korea; Seoul National University Bundang Hospital, Seoungnam-si, Kyonggi-do, Republic of Korea; Seoul National University Bundang Hospital, Seoungnam-si, Kyonggi-do, Republic of Korea; Seoul National University College of Medicine, Seoungnam-si, Kyonggi-do, Republic of Korea; Seoul National University College of Medicine, Seoungnam-si, Kyonggi-do, Republic of Korea; Seoul National University Bundang Hospital, Seoul National University College of Medicine, Seongnam-si, Kyonggi-do, Republic of Korea; Department of Laboratory Medicine, Seoul National University Bundang Hospital, Seoul, Seoul-t'ukpyolsi, Republic of Korea; Seoul National University Bundang Hospital, Seoul National University College of Medicine, Seongnam-si, Kyonggi-do, Republic of Korea

## Abstract

**Background:**

Conventional microbiological methods require several days from specimen receipt to organism identification and antimicrobial susceptibility testing (AST), delaying appropriate therapy. We evaluated an early prototype of the PhAST assay, a novel artificial-intelligence (AI)-driven single-cell imaging platform for rapid phenotypic AST.

**Figure:**
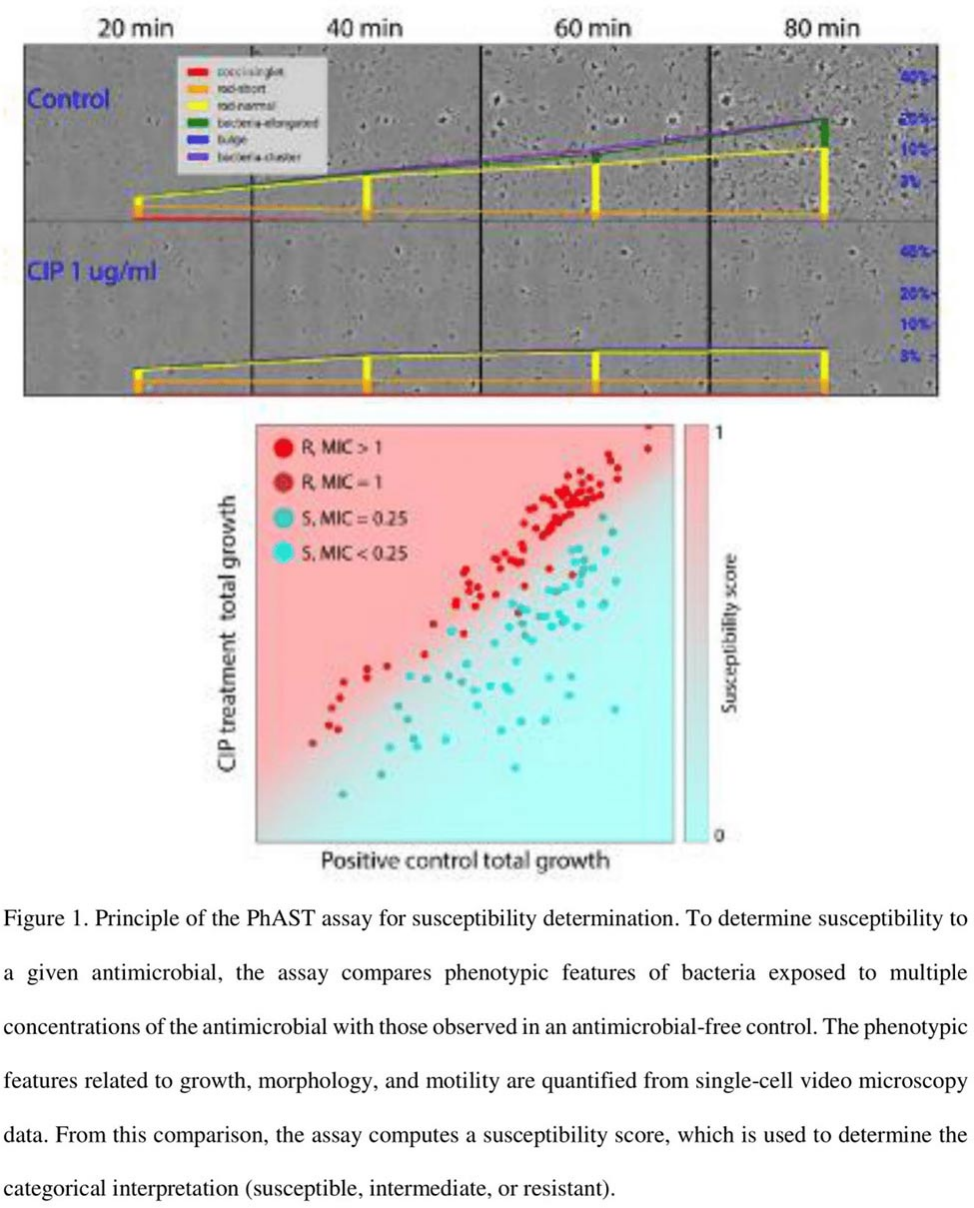

**Figure:**
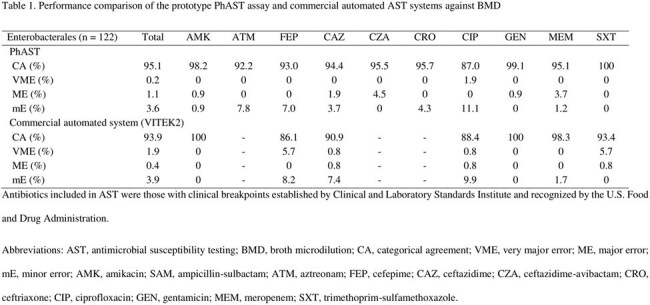

**Methods:**

The prototype PhAST assay was developed to provide bacterial identification at the group level defined by the Clinical and Laboratory Standards Institute, and phenotypic AST results were obtained in ninety minutes, directly from a positive blood culture. Susceptibility was determined by AI-based quantitative analysis of single-cell phenotypes from images and videos of antibiotic-exposed and untreated cells (Figure 1). The assay was performed on positive blood culture broth samples flagged by a routine blood culture system obtained from the clinical laboratory. Only samples containing Gram-negative bacteria were tested. We also measured the time to result (TTR) of the assay. Broth microdilution (BMD) was performed in triplicate as the reference method. Results from the commercial automated AST system (VITEK2) were also assessed against BMD.

**Results:**

A total of 122 Enterobacterales, including 69 (56.6%) *Escherichia coli*, 37 (30.3%) *Klebsiella pneumoniae*, and 6 (4.9%) *Enterobacter cloacae* complex, were identified. Resistance to third-generation cephalosporins was observed in 54 (44.3%) of the isolates, based on BMD results. The prototype PhAST assay showed a mean (± standard deviation) TTR of 117 (± 3) minutes. The assay achieved favorable results, with a categorical agreement (CA) of 95.1%, and very major error (VME), major error (ME), and minor error (mE) rates of 0.2%, 1.1%, and 3.6%, respectively (Table 1). The commercial AST system exhibited CA, ME, and mE rates of 93.9%, 0.4%, and 3.9%, respectively, with a slightly high VME rate of 1.9%.

**Conclusion:**

The prototype PhAST assay provided acceptable performance for Enterobacterales within two hours of blood culture positivity. This rapid phenotypic AST assay has significant potential to support timely clinical decision-making. Further expansion to additional organisms and antibiotics could enhance its clinical utility.

**Disclosures:**

Jeong Su Park, M.D. / Ph.D., PhAST Corp.: Advisor/Consultant|PhAST Corp.: Grant/Research Support Kyoung-Ho Song, MD, PhD, PhAST Corp.: Advisor/Consultant|PhAST Corp.: Grant/Research Support

